# Association between Alcohol Consumption and the Risk of Sarcopenia: A Systematic Review and Meta-Analysis

**DOI:** 10.3390/nu14163266

**Published:** 2022-08-10

**Authors:** Seung-Hee Hong, Yun-Jung Bae

**Affiliations:** 1 Food and Nutrition Major, Division of Food Science and Culinary Arts, Shinhan University, Uijeongbu 11644, Korea; 2 Major in Food and Nutrition, Division of Food Science and Biotechnology, Korea National University of Transportation, Jeungpyeong 27909, Korea

**Keywords:** sarcopenia, alcohol consumption, meta-analysis

## Abstract

Sarcopenia is a common disease defined as the loss of skeletal muscle mass, strength, and physical performance. Alcohol consumption is an uncertain risk factor for sarcopenia. Previous observational epidemiological studies have reported inconsistent results regarding the association between alcohol consumption and sarcopenia risk. This study aimed to investigate the association between alcohol consumption and sarcopenia. A literature review was conducted according to the Preferred Reporting Items for Systematic Reviews and Meta-Analyses (PRISMA) guidelines. We searched PubMed, EMBASE, and the Cochrane Library through April 2022 using keywords related to alcohol consumption and sarcopenia. The pooled odds ratio (OR) with a 95% confidence interval (CI) was calculated using a random effects model meta-analysis. The risk of bias of the studies was assessed using the Newcastle–Ottawa scale. Nineteen observational studies that reported 3826 sarcopenia patients among 422,870 participants were included in the qualitative analysis. Alcohol consumption was not significantly associated with sarcopenia risk (OR, 1.00; 95% CI, 0.83 to 1.20; *I*^2^ = 60.6%). Alcohol consumption resulted in a non-significant decrease in the risk of sarcopenia in men (OR, 0.70; 95% CI, 0.46 to 1.07; *I*^2^ = 0.0%) and in women (OR, 1.20; 95% CI, 0.63 to 2.30; *I*^2^ = 75.8%). The subgroup analyses by age and alcohol consumption were significantly associated with an increased the risk of sarcopenia in <65 years (OR, 2.62; 95% CI, 1.22 to 5.62; *I*^2^ = 100%). This meta-analysis of observational studies indicated that alcohol consumption was not significantly associated with sarcopenia risk. However, there are factors influencing the association between alcohol consumption and sarcopenia, such as smoking and dietary patterns. Additional study of these confounding factors is needed for the systematic analysis of the association of alcohol consumption with sarcopenia in future studies.

## 1. Introduction

Sarcopenia refers to a progressive and generalized disorder of the skeletal muscle that is associated with an increase in negative health outcomes such as falls, fractures, physical disability, and death [1]. Although sarcopenia is a common syndrome in old age, it may have a significant effect on adverse health outcomes in the elderly or patients with cancer [2]. Recent studies to identify the lifestyle and dietary factors affecting sarcopenia have been reported [3,4].

Previous studies have examined the association between sarcopenia and anti-inflammatory dietary factors among many muscle function-related dietary factors, including omega-3 fatty acids and flavonoids, which are associated with inadequate protein intake, inadequate energy intake, micronutrient deficiency, malnutrition, and inflammation [5,6,7]. The association of sarcopenia with alcohol consumption and smoking has not been clearly concluded in the study results [8,9].

Alcohol consumption causes various diseases, such as liver disease, cancer, and cardiovascular disease, possibly resulting in malabsorption of various micronutrients. In earlier studies of the association between alcohol consumption and muscle health, ethanol inhibited skeletal muscle protein synthesis in an in vivo animal model and cell experiments [10,11], and significant muscle loss and impaired protein synthesis were observed in mice that were chronically fed with ethanol [12]. In cell experiments, it has been reported that autophagy is induced by ethanol exposure, which contributes to sarcopenia [13]. Although previous human studies on alcohol consumption and sarcopenia have been conducted in various age groups and countries using an observational study design, there is no general conclusion on the association between the two. Some studies found a significant positive correlation between alcohol consumption and sarcopenia risk [14,15,16,17], whereas others reported no significant association between them [18,19,20,21].

One meta-analysis of the association between alcohol consumption and sarcopenia has been reported. The results of the meta-analysis, including 13 studies, showed that the pooled odds ratios (ORs) of sarcopenia in subjects consuming alcohol was 0.77 (95% confidence interval [CI], 0.67–0.88), indicating that alcohol consumption was not a risk factor for sarcopenia [8]. However, in that meta-analysis, there were certain limitations, such as the lack of various diagnostic criteria for sarcopenia or difficulties in alcohol consumption assessment [8]. Given the differences in the prevalence of sarcopenia and alcohol consumption patterns by sex and age, as well as the differences in socioeconomic factors that affect sarcopenia [22], a more detailed analysis by age, sex, and diagnosis criteria for sarcopenia is needed when conducting a meta-analysis. In this study, we performed a meta-analysis of the association between sarcopenia and alcohol consumption considering detailed characteristics such as age, sex, and diagnostic criteria for sarcopenia.

## 2. Methods

### 2.1. Search Strategy

This systematic review and meta-analysis was conducted in accordance with the Preferred Reporting Items for Systematic Reviews and Meta-analysis (PRISMA) guidelines [23]. We searched the MEDLINE, EMBASE, and Cochrane Library databases for eligible studies published up to April 2022. We selected both MeSH terms and keywords related to alcohol consumption and sarcopenia risk. The complete search strategy applied in PubMed was the following: (“Sarcopenia”[Mesh] OR (“sarcopenia”[TW] OR “sarcopenias”[TW] OR “skeletal muscle mass”[TW] OR “low muscle mass”[TW] OR “handgrip strength”[TW])) AND (“Alcohol Drinking”[Mesh] OR (“Drinking Alcohol”[TW] OR “Alcohol Consumption”[TW] OR “Consumption Alcohol”[TW] OR “Alcohol Intake”[TW] OR “Alcohol Intakes”[TW] OR “Intake Alcohol”[TW] OR “Alcohol Drinking Habit”[TW] OR “Drinking Habit Alcohol”[TW] OR “Habit Alcohol Drinking”[TW] OR “Habits Alcohol Drinking”[TW])).

### 2.2. Eligibility Criteria

Two reviewers independently screened the titles and abstracts, and selected full-text studies were subsequently assessed. Disagreements were resolved through consensus. Inclusion criteria for the selection of studies were based on the following: (1) all observational studies; (2) the definition of sarcopenia based on AWGS (Asian Working Group for Sarcopenia), AWGS 2019 (Asian Working Group for Sarcopenia 2019), EWGSOP (European Working Group on Sarcopenia in Older People), EWGSOP2 (European Working Group on Sarcopenia in Older People2), and FNIH (Foundation for the National Institutes of Health); and (3) information on sarcopenia with the corresponding OR and 95% CI. The following exclusion criteria were applied: (1) sarcopenia defined only by muscle quality and quantity; (2) data from the same study; (3) review, case report, and animal articles; and (4) studies that were not published in the English language. The most comprehensive study was included if it was reported in the same study.

### 2.3. Data Extraction

Data extraction was performed through two independent reviewers. Extracted information included first author, publication year, country of included participants, sarcopenia definition, body composition assessment method, sarcopenia prevalence, sex, age, definition of alcohol consumption (highest vs. lowest category), OR with 95% CI, and adjusted variables.

### 2.4. Main and Subgroup Analyses

We investigated the association between alcohol consumption (highest vs. lowest category) and the risk of sarcopenia using adjusted ORs with 95% CIs in the main analysis. We used the groups with the highest levels compared to lowest levels of alcohol consumption among the various groups in each study. We also performed subgroup analyses according to sex, age, definition criteria of sarcopenia, and geographical region of the included participants.

### 2.5. Risk of Bias Assessment

The Newcastle–Ottawa Scale (NOS) [24] for cross-sectional studies was used to assess the risk of bias. Each included study was assessed in terms of three aspects: selection, comparability, and exposure. The NOS scores ranged from zero to nine, wherein the highest possible score was nine, which reflects the lowest risk of bias. A score of five or less was classified as low quality, a score of six and seven was considered medium quality, and a score of eight and nine was classified as high quality.

### 2.6. Statistical Analysis

We used the adjusted OR and 95% CI reported in individual studies to calculate the pooled OR with 95% CI. We evaluated the heterogeneity in results across studies using Higgins *I*^2^, which measures the percentage of total variation across studies [25]. *I*^2^ was calculated as follows:

*I*^2^ = 100% × (*Q* − *df*)/*Q*

Where *Q* is Cochran’s heterogeneity statistic and *df* indicates the degrees of freedom. Negative values of *I*^2^ were set at zero; *I*^2^ lies between 0% (no observed heterogeneity) and 100% (maximal heterogeneity). An *I*^2^ value of >50% indicated substantial heterogeneity. We used a random effects model meta-analysis based on the DerSimonian and Laird method, because individual studies were conducted in different populations [26]. We also examined publication bias in the studies included in the final analysis using Begg’s funnel plot and Egger’s test. Publication bias existed if the Begg’s funnel plot was asymmetrical or the *p*-value was less than 0.05 as determined by Egger’s test. We used the Stata SE version 16 software package (StataCorp, College Station, TX, USA) for the statistical analysis.

## 3. Results

### 3.1. Selection of Relevant Studies

**Figure 1:**
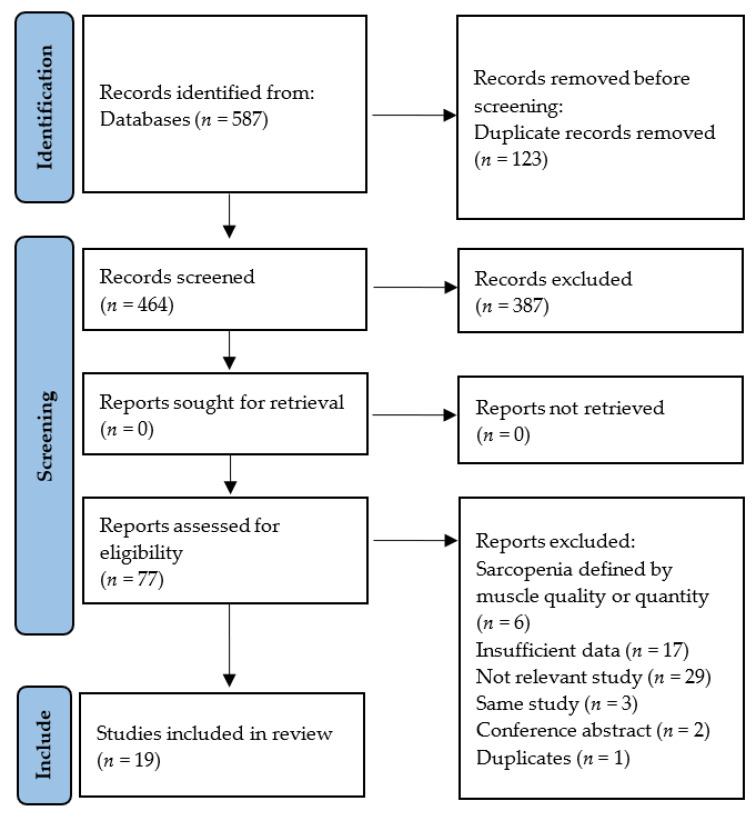
PRISMA 2020 flow chart of the article selection. Abbreviation: PRISMA, Preferred Reporting Items for Systematic Review and Meta-Analysis.

A total of 587 studies were retrieved from the preliminary search of all data (Figure 1). We excluded 123 duplicate studies and 387 studies that did not meet the selection criteria. The full text of the remaining 77 studies was reviewed, and 58 additional studies were excluded for the reasons shown in Figure 1. Finally, 19 studies [15,16,17,18,19,20,21,27,28,29,30,31,32,33,34,35,36,37,38] were included in the final meta-analysis.

### 3.2. Characteristics of Included Studies

Table 1 presents the characteristics of the studies included in the meta-analysis. Overall, 19 studies included 3826 sarcopenia patients among 422,870 participants. Nine studies were conducted in participants aged ≥65 years [18,19,21,27,28,29,33,36,37] and four studies were conducted in participants aged ≥60 years [30,31,32,34]. As for the criteria for sarcopenia definition, EWGSOP was most commonly used (six studies) [15,16,21,29,32,33], followed by EWGSOP2 (three studies) [35,36,37], AWGS 2019 (three studies) [17,18,38], AWGS (two studies) [30,31], and FNIH (one study) [19]. Eleven studies were conducted in Asia [16,17,18,21,27,29,30,31,33,37,38], five in America [15,20,28,32,34], and two in Europe [35,36].

### 3.3. Risk of Bias

Table 2 presents the individual NOS scores for each criterion in the included studies. The scores of all studies were all above five. Three studies had a score of five, fourteen studies had a score of six and seven, and two studies had a score of eight.

### 3.4. Result of the Meta-Analysis

**Figure 2:**
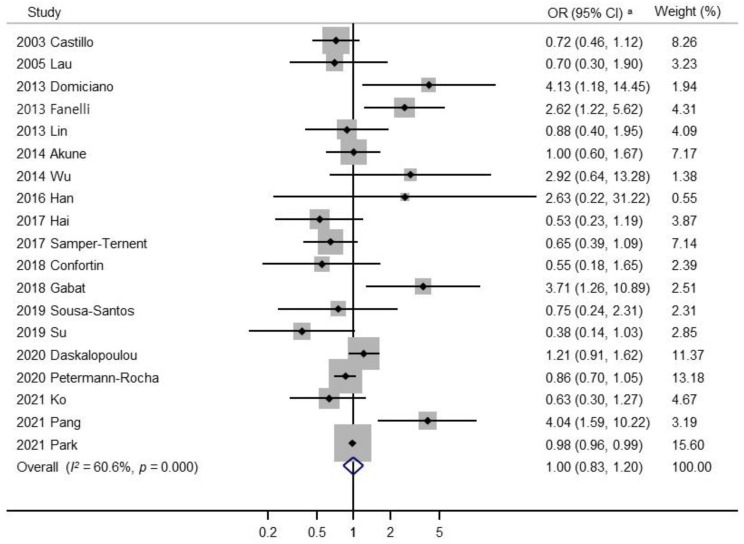
Meta-analysis of the association between sarcopenia and alcohol consumption of observational studies (*n* = 19). ^a^ Random Effects Model. Abbreviations: OR, odds ratio; CI, confidence interval [15,16,17,18,19,20,21,27,28,30,31,32,33,34,35,36,37,38].

**Figure 3:**
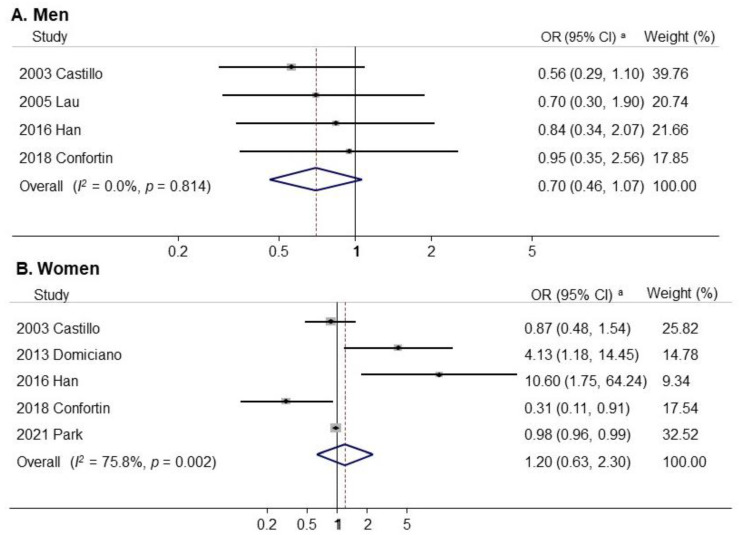
Meta-analysis of the association between sarcopenia and alcohol consumption by sex. (**A**) subgroup analysis by men; (**B**) subgroup analysis by women. ^a^ Random effects model. Abbreviation: OR, Odd Ratio; CI, Confidence Interval [20,27,28,31,34,38].

**Figure 4:**
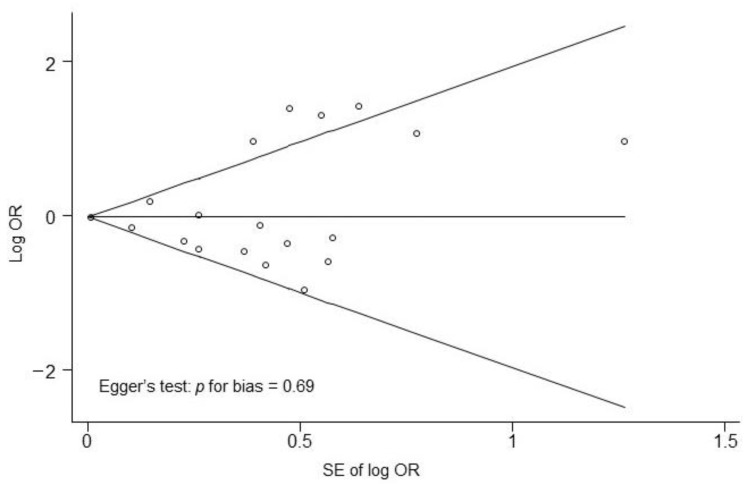
Funnel plots for identifying publication bias in the meta-analysis of observational studies. Abbreviations: OR, odds ratio; SE, standard error.

Figure 2 presents the association between alcohol consumption (highest vs. lowest) and the risk of sarcopenia in a random effects model meta-analysis of all 19 observational studies. Overall, alcohol consumption was not associated with sarcopenia risk (OR, 1.00; 95% CI, 0.83 to 1.20; *I*^2^ = 60.6%). Figure 3 presents the results of the subgroup meta-analyses by sex. Alcohol consumption was associated with a non-significant decrease in the risk of sarcopenia in men (OR, 0.70; 95% CI, 0.46 to 1.07; *I*^2^ = 0.0%; *n* = 4). In addition, alcohol consumption was not significantly associated with an increased risk of sarcopenia in women (OR, 1.20; 95% CI, 0.63 to 2.30; *I*^2^ = 75.8%; *n* = 5). Publication bias was assessed using funnel plots and Egger’s test. Begg’s funnel plot revealed a symmetric result (Figure 4). Therefore, there was no publication bias in the 19 studies (Egger’s test, *p* for bias = 0.69).

### 3.5. Subgroup Meta-Analyses

Subgroup meta-analyses were performed to determine the influence of age, definition of sarcopenia, and geographical region on the included participants. Table 3 presents the results of the subgroup analyses. In the subgroup analyses by age, alcohol consumption was significantly associated with a decreased risk of sarcopenia in ≥60 years (OR, 0.63; 95% CI, 0.42 to 0.94; *I*^2^ = 0.0%; *n* = 4), but a significantly increased risk of sarcopenia in <65 years (OR, 2.62; 95% CI, 1.22 to 5.62; *I*^2^ = 100%; *n* = 1). In the subgroup analyses according to the definition of sarcopenia, alcohol consumption was not significantly associated with an increased risk of sarcopenia in AWGS 2019 (OR, 1.24; 95% CI, 0.58 to 2.65; *I*^2^ = 80.7%; *n* = 3) and EWGSOP (OR, 1.38; 95% CI, 0.79 to 2.41; *I*^2^ = 68.5%; *n* = 6), but with a decreased risk in AWGS (OR, 0.76; 95% CI, 0.21 to 2.80; *I*^2^ = 30.9%; *n* = 2) and EWGSOP2 (OR, 0.76; 95% CI, 0.52 to 1.12; *I*^2^ = 20.1%; *n* = 3).

## 4. Discussion

To the best of our knowledge, this is the first systematic review and meta-analysis of population-based studies that focus on the association between sarcopenia and alcohol consumption. A total of 19 observational studies, representing data from 422,870 participants, of which 3826 patients had sarcopenia, were included in the meta-analysis. As a result, it was found that alcohol consumption was not significantly related to the risk of sarcopenia. However, when we conducted the subgroup meta-analyses by age group, alcohol consumption in participants aged 60 years and older decreased the risk of sarcopenia (OR, 0.63; 95% CI, 0.42–0.94).

Even with the use of subgroup meta-analysis by age group and sarcopenia diagnosis criteria, which are important factors related to the onset and diagnosis of sarcopenia, our study results should be interpreted with caution. The main results of this study were slightly different from the previously reported results of a meta-analysis performed by Steffl et al. [8]. In their meta-study, Steffl et al. analyzed the association between sarcopenia and alcohol consumption among community-dwelling elderly people aged 65 years and older in 13 cross-sectional studies, and considered baseline data from longitudinal cohort studies. The OR in the overall population was 0.77 (95% CI, 0.68–0.88), the OR in men was 0.67 (95% CI, 0.54–0.83), and the OR in women was 0.89 (95% CI, 0.73–1.08). The difference between the previous study and our study may be explained by age differences in the study, as our study performed a meta-analysis of studies of men and women aged 21 years and older in population-based groups.

Our findings indicate that the association between alcohol consumption and sarcopenia was not significant in men and women. Previous research has suggested that sex differences in the association between muscle health and alcohol consumption are linked to hormones [39]. Estrogen, a female hormone, has anabolic effects on muscle function, such as the activation and proliferation of muscle satellite cells, muscle strength, and regeneration [40,41]. The metabolism of women in the body is directly or indirectly affected by hormone secretion, and it is thought that the effects of alcohol consumption on muscle in women may vary according to changes in hormone secretion. In addition, an earlier study demonstrated that the effects of alcohol consumption on estrogen concentrations in postmenopausal women may differ depending on menopausal hormone therapy and/or the type of alcohol consumed [42]. In the future, more detailed studies examining the correlation between alcohol consumption and muscle health in women should consider hormone secretion and the characteristics of alcohol consumption.

Although alcohol consumption has been reported to induce muscle atrophy in animal models [13], the association between alcohol consumption and sarcopenia remains controversial in human studies [17,19]. Nonetheless, there are few meta-analyses of the association between alcohol consumption and sarcopenia; only one has been reported [8]. Steffl et al. [8] found that alcohol consumption was not a risk factor for sarcopenia in a meta-analysis that included only elderly individuals aged ≥65 years, and analyzed cross-sectional study data and cohort study data together. Differences in study design and study population across studies may affect the research results, which is a disadvantage of meta-analyses of observational studies. In this meta-analysis, subgroup meta-analysis according to age group was performed, and the result showed that alcohol consumption was not related to the risk of sarcopenia in the ≥65 years age group. Previous studies have reviewed the effects of alcohol consumption in elderly individuals aged 60 years and older. Among elderly women, the risk of sarcopenia was significantly higher in the high-risk alcohol consumption group, as screened by the Alcohol Use Disorders Identification Test [43], while binge drinkers with weekly or daily alcohol consumption had a higher risk of sarcopenia than social drinkers [44]. However, alcohol consumption was not a risk factor for sarcopenia when alcohol consumption was determined as either drinking or non-drinking [45]. Taken together, these findings suggest that excessive alcohol consumption may play a role as a risk factor for sarcopenia; however, the best cutoff points for alcohol consumption or frequencies to establish a significant association with sarcopenia have not been identified. In addition, a previous study reported that alcohol consumption decreases with age [46]. This may partly explain the results of the current meta-analysis study, wherein alcohol consumption was not significantly correlated with sarcopenia among the elderly aged 65 years and older, whose alcohol consumption declined.

Our meta-analysis has several limitations. First, this meta-analysis included only cross-sectional studies on alcohol–sarcopenia, and cross-sectional design studies provide a lower level of evidence than cohort studies. Second, there was a possibility of recall bias due to the data collection method used in cross-sectional studies. This meta-analysis included only cross-sectional studies because there are few reliable cohort studies on the association between alcohol consumption and sarcopenia. Third, our study data did not allow us to specify variables to measure alcohol consumption, although the type and amount of alcohol consumed by each individual were very different. Alcohol is a type of food that can be consumed and an individual’s health is greatly affected by the amount of alcohol consumed. A small amount of alcohol consumption may not be closely related to health; however, as alcohol consumption increases, the health risk increases exponentially. Therefore, it is very important to accurately estimate the amount of alcohol consumed in studies of alcohol consumption. All the studies included in the present meta-analysis asked participants to self-report their alcohol consumption using survey questions regarding alcohol consumption variables. Nevertheless, given the previous study by Del Boca et al. [47], in which self-report methods were shown to have a higher degree of similarity to actual drinking episodes than other approaches, we believe that the validity of this study is adequate.

Our study has several strengths. This meta-analysis contributes to our understanding of the effects of alcohol consumption on sarcopenia by conducting a subgroup meta-analysis according to sex, age, diagnostic criteria for sarcopenia, and region. As sarcopenia accelerates with age, and the patterns of alcohol consumption change with age, this study analyzed the association between alcohol consumption and sarcopenia by subgroups based on age. Moreover, there are various diagnostic criteria for sarcopenia, such as AWGS, AWGS 2019, EWGSOP, and EWGSOP2. There are many diagnostic indicators of sarcopenia, including grip strength, physical performance, five-time chair stand test, and muscle mass (by bioimpedance or dual-energy X-ray absorptiometry method), and cut-off values for muscle mass for evaluation of muscle mass loss differ by race. Therefore, the diagnostic criteria for sarcopenia are diverse and continue to be updated. In this study, to clarify the association between alcohol consumption and sarcopenia, we analyzed the association between alcohol consumption and subgroups according to the diagnostic criteria for sarcopenia and found no significant association. Age, dietary patterns, socioeconomic factors, and physical activities are expected to influence the association between sarcopenia and alcohol consumption. One of the characteristics of original papers in this meta-analysis is the adjustment for various factors such as age, smoking status, physical activity levels, body mass index (BMI), and education levels (Table 1).

In conclusion, the results of this meta-analysis of 19 studies showed that alcohol consumption was not significantly associated with sarcopenia risk. However, the association between sarcopenia and alcohol consumption may differ according to age. An implication of this is that the association between alcohol consumption and sarcopenia may be different in a particular group by sex and age. The effects of alcohol use on health-related outcomes have been analyzed using various variables for alcohol consumption, such as alcohol consumption, frequency, and amount of alcohol consumed. However, there are few studies on alcohol consumption and sarcopenia considering various alcohol use variables. Future studies on alcohol–sarcopenia are needed to systematically assess alcohol consumption in various subjects. Considering the variety of diagnostic indicators of sarcopenia, such as grip strength and physical performance along with appendicular skeletal muscle mass, detailed studies on the association between alcohol consumption and the various sarcopenia indicators are anticipated.

## Figures and Tables

**Table 1:** Characteristics of the included studies.

Study	Country, Study Design	Definition of Sarcopenia	Body Composition	Participants (Sarcopenia/ No Sarcopenia)	Sex (M/W)	Age (Years)	Expose (Highest Category)	Reference (Lowest Category)	OR (95% CI)	Adjusted Variables
2003 Castillo [20]	USA, cross-sectional study	FFM of ≥2.0 SDs below the mean of a young reference group	BIA	1700 (102/1598)	694M/1006W	55–98	≥181.0 g/week for men, ≥120.5 g/week for women	<181.0 g/week for men, <120.5 g/week for women	0.72 (0.46–1.12)	Age, exercise, smoking
2005 Lau [27]	Hong Kong, cross-sectional study	Total adjusted skeletal muscle mass two SDs or more below the mean of young men	DXA	173 (32/141)	173M	≥70	Daily	Never	0.70 (0.30–1.90)	Age
2013 Domiciano [28]	Brazil, cross-sectional study	Baumgartner’s criteria (ASM/height^2^ is less than 5.45 kg/m^2^)	DXA	611 (23/588)	611W	≥65	Three or more units daily	No	4.13 (1.18–14.45)	Age
2013 Fanelli [15]	USA, cross-sectional study	EWGSOP	DXA	2176 (139/2037)	945M/1231W	30–64	Alcohol drink cluster	Healthy pasta/rice reference cluster	2.62 (1.22–5.62)	Sex, race, age, socioeconomic status
2013 Lin [29]	Taiwan, cross-sectional study	EWGSOP	DXA	761 (99/662)	407M/354W	≥65	Current	Never	0.88 (0.40–1.95)	Age, sex, marital status, regular exercise habit, comorbidity status (diabetes mellitus, stroke, heart disease, cataract, fall history)
2014 Akune [21]	Japan, cross-sectional study	EWGSOP	BIA	1000 (129/871)	349M/651W	≥65	Yes	No	1.00 (0.60–1.67)	Age, sex, BMI
2014 Wu [33]	Taiwan, cross-sectional study	EWGSOP	BIA	549 (70/479)	285M/264W	≥65	Yes	No	2.92 (0.64–13.28)	None
2016 Han [31]	China, cross-sectional study	AWGS	BIA	1069 (99/970)	437M/533W	≥60	Daily	Never or former	2.63 (0.22–31.22)	Age, BMI, widowed, living alone, illiteracy, farming, diabetes, peptic ulcer, pulmonary disease
2017 Hai [30]	China, cross-sectional study	AWGS	BIA	834 (88/746)	415M/419W	≥60	Drinking ≥2/week	Not drinking	0.53 (0.23–1.19)	Gender, age, educational level, diabetes, hypertension, heart disease, stroke, MMSE score, GDS score
2017 Samper-Ternent [32]	Colombia, cross-sectional study	EWGSOP	DXA	1442 (166/1276)	562M/880W	≥60	≥1 glass per day	No alcohol consumption	0.65 (0.39–1.09)	Age, sex, education, comorbidities, MMSE score, GDS score, IADL disability, ADL disability, smoking
2018 Confortin [34]	Brazil, cross-sectional study	Baumgartner’s criteria (ASMI: <7.26 kg/m^2^ for men and <5.5 kg/m^2^ for women)	DXA	598 (126/472)	207M/391W	≥60	Continued consuming or started consuming alcohol	Continued not consuming or stopped consuming alcohol	0.55 (0.18–1.65)	Age, schooling, income, marital status, family arrangement, smoking, physical activity, social support, self-rated health
2018 Gabat [16]	Philippine, cross-sectional study	EWGSOP	FBCM	164 (10/154)	37M/127W	≥40	Yes	No	3.71 (1.26–10.89)	Controlling possible confounders
2019 Sousa-Santos [36]	Portugal, cross-sectional study	EWGSOP2	MAMC	1500 (66/1434)	628M/872W	≥65	Women >1/day: men >2/day	None	0.75 (0.24–2.31)	Sex, age, residential status, regional area, educational level, marital status, BMI, physical activity level, undernutrition status
2019 Su [37]	Japan, cross-sectional study	EWGSOP2	BIA	310 (25/285)	89M/221W	≥65	Consumes alcohol	None	0.38 (0.14–1.03)	None
2020 Daskalopoulou [19]	LMICs., Multicenter population study	FINH	Body fat percent (%BF)	8694 (-/-)	8694MW	≥65	1–14 units/week for women and 1–21 units/week for men	No/heavy	1.21 (0.91–1.62)	Dementia, depression, diabetes, stroke
2020 Petermann-Rocha [35]	UK, cross-sectional study	EWGSOP2	BIA	396283 (1678/394605)	187046M/209237W	38–73	Higher	Lower	0.86 (0.70–1.05)	Age, sex, deprivation, education attainment
2021 Ko [18]	Taiwan, cross-sectional study	AWGS 2019	BIA	500 (138/362)	235M/265W	≥65	Yes	No	0.63 (0.30–1.27)	Sex, institutionalization, age, BMI, albumin, hemoglobin, HDL-C levels, history of cardiovascular disease, education level
2021 Pang [17]	Singapore, cross-sectional study	AWGS 2019	DXA	536 (132/404)	226M/310W	21–90	Yes	No	4.04 (1.59–10.22)	None
2021 Park [38]	Korea, cross-sectional study	AWGS 2019	DXA	3970 (704/3266)	3970W	≥40	Yes	No	0.98 (0.96–0.99)	None

Abbreviations: M, men; W, women; OR, odds ratio; CI, confidence interval; FFM, fat-free mass; BIA, bioelectric impedance analysis; SD, standard deviation; DXA, dual-energy X-ray absorptiometry; EWGSOP, European Working Group on Sarcopenia in Older People; EWGSOP2, European Working Group on Sarcopenia in Older People2; AWGS, Asian Working Group for Sarcopenia; AWGS 2019, Asian Working Group for Sarcopenia 2019; ASM, appendicular skeletal mass; ASMI, appendicular skeletal mass index; FBCM, Fresenius body composition monitor; MAMC, mid-arm muscle circumference; LMICs, low-and middle-income countries; FINH, Foundation in the National Institutes of Health; BMI, body mass index; MMSE, mini-mental state examination; GDS, geriatric depression scale; IADL, instrumental activities of daily living; ADL, activities of daily living; HDL-C, high density lipoprotein cholesterol.

**Table 2:** Quality assessment of the included studies using the NOS.

Study	Selection				Comparability		Exposure			Total
	1	2	3	4	5A	5B	6	7	8	
2003 Castillo [20]	1	1	1	1	1	1	0	1	0	7
2005 Lau [27]	1	1	1	1	0	0	0	1	0	5
2013 Domiciano [28]	1	1	1	1	1	0	0	1	0	6
2013 Fanelli [15]	1	1	1	1	1	1	0	1	0	7
2013 Lin [29]	1	1	1	1	0	0	0	1	0	5
2014 Akune [21]	1	1	1	1	1	1	0	1	1	8
2014 Wu [33]	1	1	1	1	0	0	0	1	1	6
2016 Han [31]	1	1	1	1	1	1	0	1	0	7
2017 Hai [30]	1	1	1	1	1	1	0	1	0	7
2017 Samper-Ternent [32]	1	1	1	1	1	1	0	1	0	7
2018 Confortin [34]	1	1	1	1	1	1	0	1	1	8
2018 Gabat [16]	1	1	1	1	1	1	0	1	0	7
2019 Sousa-Santos [36]	1	1	1	1	1	1	0	1	0	7
2019 Su [37]	1	1	1	1	0	0	0	1	0	5
2020 Daskalopoulou [19]	1	1	1	1	0	1	0	1	1	7
2020 Petermann-Rocha [35]	1	1	1	1	1	1	0	1	0	7
2021 Ko [18]	1	1	1	1	1	1	0	1	0	7
2021 Pang [17]	1	1	1	1	1	1	0	1	0	7
2021 Park [38]	1	1	1	1	1	1	0	1	0	7

Newcastle–Ottawa Scale (NOS) for cross-sectional studies (Yes = 1, No = 0). 1: Adequate definition of cases. 2: Cases are consecutive or obviously representative. 3: Selection of controls. 4: Definition of controls. 5A: Comparability of cases and controls on the basis of the design or analysis adjusted for age. 5B: Comparability of cases and controls on the basis of the design or analysis adjusted for additional factors. 6: Ascertainment of exposure. 7: Same method of ascertainment for participants. 8: Non-response rate.

**Table 3:** Subgroup meta-analyses in relation to sarcopenia and alcohol consumption.

Factors	Number of Studies	Summary OR (95% CI)	Heterogeneity, *I*^2^ (%)
Age			
40 years and older [16,20,38]	3	1.07 (0.65–1.74)	74.0
60 years and older [30,31,32,34]	4	0.63 (0.42–0.94)	0.0
65 years and older [15,18,19,21,28,29,33,36,37]	9	0.97 (0.69–1.36)	44.8
65 years and younger [27]	1	2.62 (1.22–5.62)	100
Definition of sarcopenia			
AWGS [30,31]	2	0.76 (0.21–2.80)	30.9
AWGS 2019 [17,18,38]	3	1.24 (0.58–2.65)	80.7
EWGSOP [15,16,21,29,32,33]	6	1.38 (0.79–2.41)	68.5
EWGSOP2 [35,36,37]	3	0.76 (0.52–1.12)	20.1
Region			
America [15,20,28,32,34]	5	1.12 (0.58–2.16)	75.7
Asia [16,17,18,21,27,29,30,31,33,37,38]	11	1.03 (0.74–1.45)	60.0
Europe [35,36]	2	0.86 (0.70–1.05)	0.0

Abbreviations: OR, odds ratio; CI, confidence interval; AWGS, Asian Working Group for Sarcopenia; AWGS 2019, Asian Working Group for Sarcopenia 2019; EWGSOP, European Working Group on Sarcopenia in Older People; EWGSOP2, European Working Group on Sarcopenia in Older People2.

## Data Availability

Not applicable.

## Abbreviations

AWGS	Asian Working Group for Sarcopenia
AWGS 2019	Asian Working Group for Sarcopenia 2019
CI	confidence interval
EWGSOP	European Working Group on Sarcopenia in Older People
EWGSOP2	European Working Group on Sarcopenia in Older People2
FNIH	Foundation for the National Institutes of Health
OR	odds ratio
NOS	Newcastle–Ottawa Scale
PRISMA	Preferred Reporting Items for Systematic Reviews and Meta-analysis
